# A New Challenging Strategy in the Prevention and Management of Tumor Lysis Syndrome in Patients with Chemo-Sensitive Hematological Malignancies

**DOI:** 10.1155/2019/3740547

**Published:** 2019-05-19

**Authors:** Ammar ELJack, Mohamad El Abdallah, Kadhim Al-Banaa, Kashif Chaudhry, Faisal Musa

**Affiliations:** ^1^Department of Internal Medicine, Beaumont Hospital-Dearborn, 18101 Oakwood, Dearborn, MI 48124, USA; ^2^Department of Nephrology, Beaumont Hospital-Dearborn, Dearborn, MI, USA; ^3^Department of Hematology and Oncology, Beaumont Hospital-Dearborn, Dearborn, MI, USA

## Abstract

**Introduction:**

Tumor lysis syndrome (TLS) is a metabolic derangement that results from rapid destruction of cells. It happens frequently in cancers receiving chemotherapy, particularly hematological malignancies. It can lead to death in severe cases. Tumor lysis syndrome that leads to acute renal failure requiring dialysis and/or ICU admission can be associated with a higher rate of complications and mortality.

**Case Report:**

We present a 24-year-old male patient with Burkitt's lymphoma. After receiving one cycle therapy, he developed severe kidney injury from TLS. We initiated renal replacement therapy soon after his admission to the ICU, with marked response to therapy. This led to early discharge from the ICU.

**Conclusion:**

Early initiation of renal replacement therapy after TLS-AKI can improve the severity of AKI and hasten recovery and prevent complications. This can lead to earlier discharge from the hospital and better outcomes.

## 1. Introduction

Tumor lysis syndrome (TLS) is a serious complication of chemotherapy in rapidly progressive cancer. It presents clinically with nausea, vomiting, fatigue, acute kidney injury, seizure, arrhythmia, and even death. It is caused by the release of electrolytes from the damaged cells. Characteristic electrolyte disturbances include hyperuricemia, hyperkalemia, hyperphosphatemia, and hypocalcemia [1]. The National Comprehensive Cancer Network (NCCN) guidelines recommended prophylaxis and treatment with aggressive hydration and control of hyperuricemia with allopurinol (xanthine oxidase inhibitor) 2-3 days prior to chemotherapy or rasburicase (urate oxidase) which is highly effective in prevention and treatment of TLS. It is indicated for high-risk patient, patient with adequate hydration not possible to achieve, urgent therapy in high-bulk patient, and acute kidney injury [2].

Acute kidney injury (AKI) may frequently complicate TLS. It is caused by precipitation of uric acid, calcium phosphate, or hypoxanthine in the renal tubules [3]. It may lead to renal failure requiring renal replacement therapy (RRT) in its most severe forms.

## 2. Case Scenario

**Figure 1:**
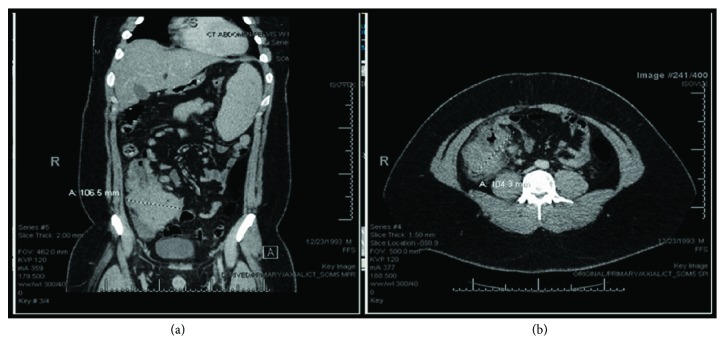
CT scan of the abdomen ((a) axial section, showed large 10 × 10 cm ileocolic mass, lymphadenopathy at the right colic area, and splenomegaly; (b) horizontal section).

A 24-year-old male with no significant past medical history presented to ED with gradually worsening abdominal pain for one month. His symptoms were associated with anorexia, black tarry stools, intermittent fever, sweating, vomiting, dizziness, and significant unintentional weight loss (40 lbs in 3 months). On admission, his vital signs were stable. He was anxious and clammy, and his physical exam was significant for splenomegaly. Initial blood work showed leukocytosis with a white cell count of 33.9 K/Ul, hemoglobin was 15.3 g/dl, platelet count was 67 K/Ul with blast cells of 30%. The liver panel showed aspartate aminotransferase (AST) of 161 IU/l, alanine aminotransferase (ALT) of 65 IU/l, and alkaline phosphatase (ALP) of 64.3 IU/l. Total bilirubin was 0.9 mg/dl. Lactate dehydrogenase (LDH) was 6000 U/l, and uric acid was 11.3 mg/dl. Urea was 14 mg/dl, and serum creatinine was 1.24 mg/dl. Na was 139 mmol/l, K was 3.9 mmol/l, and Ca was 9.6 mmol/l. An abdominal CT scan showed large ileocolic mass with enlarged lymphadenopathy at the right colic area (Figure 1).

**Figure 2:**
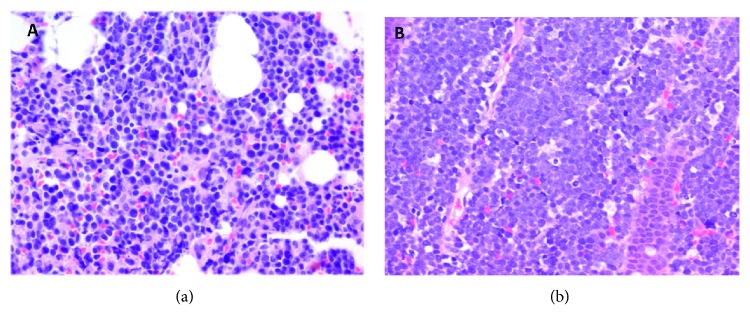
(a) Bone marrow biopsy with 400x H&E stain showed diffuse infiltrate and characterized by medium-to-large cells with irregular nuclear contour, prominent nucleoli, and cytoplasmic vacuoles. Brisk mitosis identified. (b) Colon biopsy with 400x H&E stain showed intermediate-sized lymphoid cells with scattered histiocytes imparting a “starry sky” pattern.

Peripheral smear showed immature cells with increase blast suggestive of acute leukemia. Biopsy of the colonic mass showed lymphoma. Flow cytometry confirmed the diagnosis of Burkitt's lymphoma. CT head, neck, and thorax revealed no other lymph node involvement (Figure 2).

**Figure 3:**
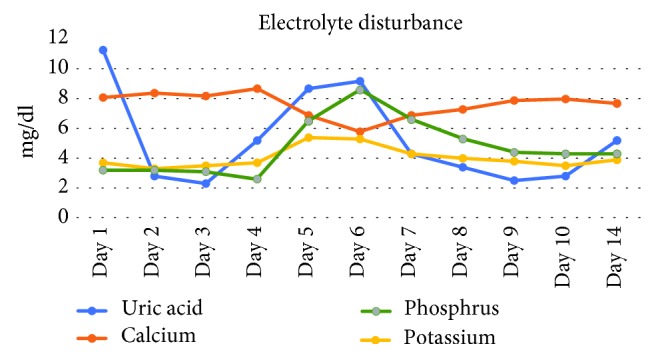
Electrolyte disturbance in TLS patient over the days since hospital admission.

The patient was diagnosed with stage IV Burkitt's lymphoma with leukemic phase. Treatment was initiated with R hyper-CVAD (hyper-fractionated cyclophosphamide, vincristine, doxorubicin, and dexamethasone); he received one cycle of part A, then switched to another treatment regimen DA EPOCH-R (dose-adjusted etoposide, prednisone, vincristine, cyclophosphamide, doxorubicin, and rituximab), and prophylactic intrathecal chemotherapy (methotrexate, cytarabine, and hydrocortisone). On day 2 of admission, the patient developed TLS-AKI. His glomerular filtration rate (GFR) dropped to 22 ml/min/1.73 m^2^ and serum creatinine increased to 4 mg/dl (Figure 3).

Treatment was started with aggressive hydration, rasburicase, and allopurinol. On day 5, all of the previous measures failed, and his kidney functions were deteriorated, so renal replacement therapy (RRT) was initiated and continued for a total of 14 days. He responded well to therapy and was discharged from the hospital after 25 days of management with recommendations for outpatient follow-up.

## 3. Discussion

The overall incidence of TLS was reported at 4.4% in two large multicenter studies of non-Hodgkin lymphoma (NHL). Of these, TLS occurred in 8.4% of the patients diagnosed with Burkitt's lymphoma/leukemia or B-cell acute lymphocytic leukemia (B-ALL) [4].

In a large retrospective study in the United States, the nation-wide estimate of patients admitted with TLS was 22,785. 12.8% of them developed acute kidney injury requiring dialysis (AKI-D) [5]. AKI-D patients have higher mortality (41.9% vs. 19.1%; *P* < 0.01) and longer hospital stay than patients with AKI without dialysis requirement (19 vs. 14.9 days; *P* < 0.01) [5]. In a single-center study at Saint-Louis University Hospital in France that included 63 patients, the adjusted mortality at hospital discharge was higher in patients with TLS who developed AKI (odds ratio: 10.41; 95% confidence interval: 2.01-19.17; *P* = 0.005). Interestingly, RRT was performed in 17 patients without AKI and in 31 patients with TLS-induced AKI [6].

TLS-induced AKI may decrease the probability of getting a long-term remission of the malignancy; therefore, prevention of the development of AKI plays an essential role in the management of TLS, and consequently, primary malignancy outcome [6, 7]. In patients with hematological malignancies who require chemotherapy, anticipation and early recognition of TLS and intervention play crucial roles in the treatment and prevention of complications. Although RRT by self cannot prevent AKI from occurrence in a patient with TLS, we challenge that early initiation of RRT could play a pivotal role in preventing complications associated with TLS, even in early stages of AKI. Further clinical trials are needed to evaluate early intervention using RRT to assess prognosis as well as mortality and morbidity in patients with chemo-sensitive hematological malignancies who developed TLS.
